# The Lost Art of Prescribing

**Published:** 1909-06-26

**Authors:** 


### THE LOST ART OF PRESCRIBING.

It is a venerable grumble among physicans of the older school that the art of therapeutics is decaying because the younger generation is extremely ignorant of materia medica and remarkably inefficient in the matter of prescribing elegant mixtures. The result, it is averred, is that proprietary drugs are acquiring an increasing vogue to the detriment both of the patient and the practitioner. A recent contributor to the *Medical Record* has made this thesis the text of an address upon the proper teaching of therapeutics in medical schools. "I have maintained for years," he says, "that the best way to do away with nostrums is to give our medical students thorough courses in materia medica, medical pharmacy, pharmacology, and therapeutics. The way to abolish proprietary medicines is to teach medical students how to prescribe, and acquaint them with the physiological and therapeutic action of drugs. They should be taught how to write or compound prescriptions that would be palatable and agreeable, compatible, yet so associated or combined as to meet the indications for which the prescription is intended in a scientific manner."

With due submission to the ripe experience of these praisers of the past we take leave to question the deduction while admitting the premiss. It is past question, we believe, that the younger generation of medical men is far behind its predecessors in the matter of prescription-writing: a thing in itself to be regretted. But the march of events which has evolved this state of affairs has not been without its compensations, to the patient at least. It may be granted, from the professional point of view, that the old-fashioned "grapeshot" prescription, as it has been irreverently called, was a triumph of art, and that to combine a dozen medicinal substances in one draught so skilfully that it should neither precipitate, nor explode, nor revolt the patient's stomach, was no mean achievement. But to say that the loss of this faculty has invited the inroads of proprietary medicines is an assumption not only unproved, but probably incorrect. To us at least such a proposition seems an argument of the *post, ergo propter* variety, for the following reasons. Fifty years ago the Pharmacopœia was largely composed of crude drugs, for pharmaceutical chemistry was relatively in its childhood, and standardisation of drugs was not attempted. With the rough materials at his disposal the physician of the time no doubt did wonders in the way of obscuring nauseous qualities and compounding imposing formulæ. Time slipped away and presently there arose a generation of chemists who were not content with the old crude drugs, but set to work to standardise them and isolate their active principles. From this stage it was but a step to the subversion of the old-fashioned draught and its replacement by preparations less bulky and more convenient, and at the same time more pleasant to take. For with all their boasted skill in compounding elegant mixtures the "grapeshot" school seems to have left a tradition among the contemporary laity that draughts even in those days were not grateful to the palate. We have to consider, then, on the one hand a time in which drugs were crude, unstandardised, very variable in strength, and administered in a form which, if as palatable as it was possible to make it, was nevertheless inconvenient and distasteful: on the other we have an epoch in which the active principles of those drugs can be obtained pure, standardised, vouched for in both these respects by chemical firms of high scientific reputation, and withal convenient and easy to administer. Can it be wondered at that the medical practitioner of the present day finds himself driven, even against his material interest, to give the proved and pleasant forms of drug which the patient knows well enough are on the market, rather than to spend his time in learning the *finesse* of prescribing the antiquated remedies of the British Pharmacopœia.

The truth is that our Pharmacopœia is an anachronism. It contains, of course, plenty of old and well-tried friends, but they are almost swamped in rubbish. When a person has travelled by an express train it is idle to assure him that a coach is the best way of getting about, and still more idle to complain, when he refuses to go back to coaching, that his tiresome choice is due to the fact that coachmen have forgotten how to drive. The British Pharmacopœia does not meet the needs of the present day, and in consequence it goes to the wall. No one is responsible for this except those whose business it is to keep the Pharmacopœia level with the march of civilisation. This they have neglected to do and the result is what we see.

